# A novel homozygous mutation in *TRAPPC9* gene causing autosomal recessive non-syndromic intellectual disability

**DOI:** 10.1186/s12920-022-01354-1

**Published:** 2022-11-08

**Authors:** Mutaz Amin, Cedric Vignal, Esraa Eltaraifee, Inaam N. Mohammed, Ahlam A. A. Hamed, Maha A. Elseed, Arwa Babai, Iman Elbadi, Doua Mustafa, Rayan Abubaker, Mohamed Mustafa, Severine Drunat, Liena E. O. Elsayed, Ammar E. Ahmed, Odile Boespflug-Tanguy, Imen Dorboz

**Affiliations:** 1 Faculty of Medicine, Al-Neelain University, Khartoum, Sudan; 2 INSERM UMR 1141 PROTECT, Université Paris Diderot- Sorbonne Paris Cité, Paris, France; 3 Unité de Génétique Moleculaire, Departement de Genetique Médicale, APHP, Hopital Robert-Debré, 75019 Paris, France; 4 Faculty of Medicine, University of Khartoum, Khartoum, Sudan; 5 National University Biomedical Research Institute, National University, Khartoum, Sudan; 6 Department of Basic Sciences, College of Medicine, Princess Nourah Bint Abdulrahman University, P.O. Box 84428, Riyadh, 11671 Saudi Arabia; 7 Neuropediatrics and Metabolic Disorders Department, Reference Center for Leukodystrophies and Rare Leukoencéphalopathies (LEUKOFRANCE), CHU APHP Robert-Debré, Imen DORBOZ: INSERM U1141, Hopital Robert Debre, 48 boulevard Serurier, 75019 Paris, France

**Keywords:** Autosomal recessive, Intellectual disability, TRAPPC9, Novel, Sudan

## Abstract

**Background:**

The etiology of intellectual disabilities is diverse and includes both genetic and environmental factors. The genetic causes of intellectual disabilities range from chromosomal aberrations to single gene disorders. The *TRAPPC9* gene has been reported to cause autosomal recessive forms of intellectual disabilities in 56 patients from consanguineous and non-consanguineous families around the world.

**Methods:**

We analyzed two siblings with intellectual disability, microcephaly and delayed motor and speech development from a consanguineous Sudanese family. Genomic DNA was screened for mutations using NGS panel (NextSeq500 Illumina) testing 173 microcephaly associated genes in the Molecular Genetics service in Robert Debre hospital in Paris, France.

**Results:**

A novel homozygous mutation (NM_031466.7 (TRAPPC9):c.2288dup, p. (Val764Glyfs*7) in exon 14 of *TRAPPC9* gene was found in the two patients. The mutation was predicted to cause nonsense mediated decay (NSMD) using SIFT prediction tool. The variant has not been found in either gnomAD or Exac databases. Both parents were heterozygous (carriers) to the mutation.

**Conclusion:**

This is the first study to report patients with *TRAPPC9*-related disorder from Sub-Saharan Africa.

## Background

Intellectual disabilities (ID) are a heterogeneous group of disorders that present with variable severity of cognitive impairment which can be associated with other behavioural, syndromic or dysmorphic features [1]. There are currently more than 700 known rare genetic diseases that can present with various forms of intellectual disabilities and can be inherited as autosomal recessive, autosomal dominant, X-linked or mitochondrial [2]. Autosomal recessive forms of intellectual disabilities are relatively rare and account for less than 12% of cases of intellectual disabilities [3]. However, they are particularly more common in consanguineous communities as in the Middle East [4–12].

Intellectual disability-obesity-brain malformations-facial dysmorphism syndrome (ORPHA: 352,530) is a very rare form of autosomal recessive intellectual disability characterized by moderate to severe intellectual impairment, epilepsy, microcephaly, variable dysmorphic features and obesity [13]. The disease is caused by loss of function mutations in *TRAPPC9* gene which is located in chromosome 8q24.3 and has 23 exons [6]. It encodes a protein that has important roles in brain development and functions as an activator of NF-kappa-B through increased phosphorylation of the IκB kinase (IKK) complex [5]. The clinical spectrum related to *TRAPPC9* mutations also include non-syndromic intellectual disability [14] autism [15] and severe developmental delay [16].

So far, mutations in *TRAPPC9* gene have been reported in very few families around the world [4–12, 14, 15, 17–25] but none from Sub-Saharan Africa. In this study, we reported a novel homozygous mutation in *TRAPPC9* gene causing autosomal recessive intellectual disability in a consanguineous family from Sudan.

## Methods

Two affected siblings born to consanguineous parents were studied. Patient one (aged 6 years) and patient two (aged 4 years) were both males and outcomes of normal uncomplicated vaginal delivery. Both patients presented with severe delay in motor development and total lack of speech up to the time of assessment (6 and 4 years respectively) (patient 1 started to walk at age 5 years and patient 2 at 2.5 years). Obesity was noted in the first few months of life followed by acquired microcephaly noted after 1 year of age. Both patients had severe learning difficulty (they were unable to attend school) and cognitive impairment. In addition, they had behavioral abnormalities (hyperactivity, irritability, biting and frequent nocturnal awakening). Patient two also had epilepsy. On examination, they had microcephaly (< 3 SD), hypotonia and hyporeflexia (Table 1.).

**Table 1:** Clinical features of patients with mutations in *TRAPPC9* gene

No	Reported patients	This study	
	56	Patient 1	Patient 2
Consanguinity	15/20 (75%)	+	+
Age in years (mean)	12.2	6	4
Sex (M/F)	19/27	+	+
Intellectual disability	53/53 (100%)	+	+
Microcephaly	42/47 (89%)	+	+
Dysmorphism	24/39 (61%)	−	−
Delayed motor and speech development	54/54 (100%)	+	+
Autistic features	7/25 (28%)	−	−
Epilepsy	8/39 (18%)	−	+
Obesity	13/28 (46%)	+	+
Behavioral abnormalities	15/18 (83%)	+	+
MRI findings			
Brain atrophy	11/14 (78%)	+	+
White matter changes	19/22 (86%)	+	+
Thin corpus callosum	24/26 (92%)	+	+

**Fig. 1:**
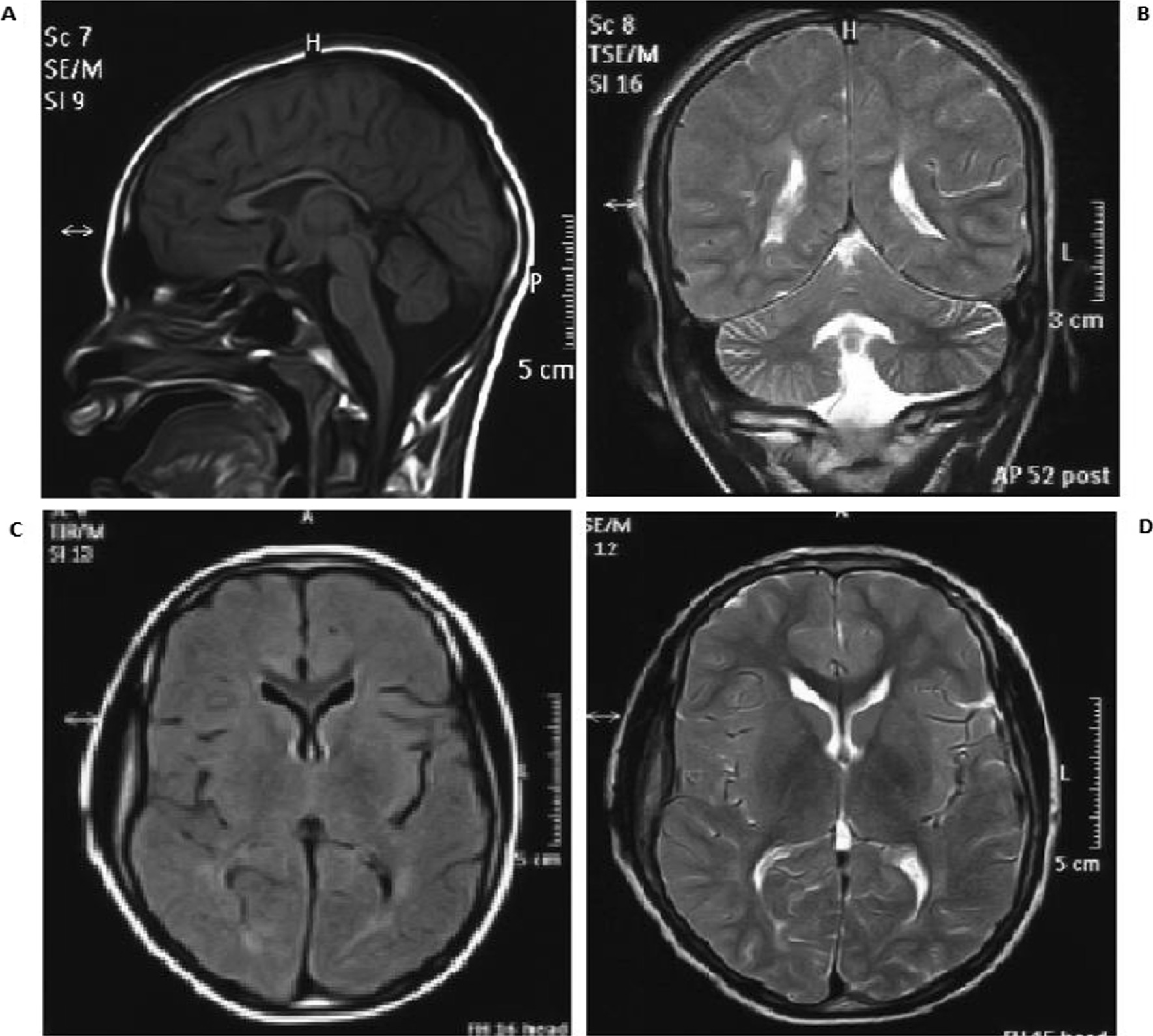
Brain MRI (T1) of patient one showing mild cortical and cerebellar atrophy, white matter abnormality and thin corpus callosum (**A**: T1 (sagittal section),** B**: T2 (coronal section),** C**: T1 (cross section),** D**: T2 (cross section))

Brain MRIs of both patients revealed cortical and cerebellar atrophy, periventricular white matter changes, thin corpus callosum, dilatation of ventricles, and hyperintensity of posterior limb of internal capsule (Fig. 1.).

### Targeted gene panel testing

Two ml of saliva was collected from patients and other healthy family members using DNA Oragene Saliva kits (DNA Genotek Inc., Ottawa, ON, Canada). DNA extraction was done according to the prepIT.L2P manual protocol provided by the manufacturer. Genomic DNA was screened for mutations using NGS panel (NextSeq500 Illumina) testing 173 microcephaly associated genes in the Molecular Genetics service in Robert Debre hospital in Paris, France. Library was prepared using Custom SureSelectQXT (GC_V3) Agilent. Burrows-Wheeler Aligner algorithm (BWA) was used to align genomic DNA sequence to human reference genome GRCh37 (hg19) with > 99.5% coverage and minimum depth of 20X. Variants were classified using Bench lab NGS Cartagenia v5.0.1.

## Results

A novel homozygous mutation (NM_031466.7 (TRAPPC9):c.2288dup, p. (Val764Glyfs*7) in exon 14 of *TRAPPC9* gene was found in the two patients. The mutation was a frameshift mutation leading to premature stop codon and was predicted to be deleterious using ENTPRISE-X [26] causing nonsense mediated decay (NSMD) as predicted by the SIFT tool [27]. The variant has not been found in either gnomAD or Exac databases. Parents were screened for the mutation using the same panel NGS analysis described in Methods and both were heterozygous (carriers) to the mutation. No other potentially deleterious variants were found in genes involved in neurodevelopmental disorders.

## Discussion

**Fig. 2:**
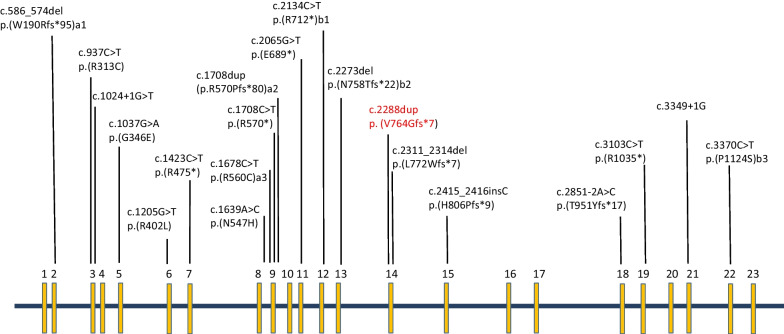
Reported mutations in *TRAPPC9* gene (NM_031466.7 transcript). Our reported mutation is shown in red. **a**, **b** Compound heterozygous mutations

The development of the human brain is very complex and involves a cascade of reactions controlled by significant number of genes [28]. In this study, we reported a novel homozygous mutation in *TRAPPC9* gene causing autosomal recessive intellectual disability in a Sudanese family. The mutation reported in our study is a one base pair duplication in exon 14 that results in a premature stop codon and predicted to cause nonsense mediated decay in TRAPPC9*-*mRNA. All *TRAPPC9* mutations reported so far are loss of function mutations (nonsense, frameshift splice site or inframe insertions/deletions) (Fig. 2.) and there was no genotype–phenotype correlation [4–7, 9–12, 18].

*TRAPPC9*-related intellectual disability is an autosomal recessive disease with particular overrepresentation in consanguineous communities [4–12]. To our knowledge, only 56 patients were reported to have mutations in *TRAPPC9* gene [4–12, 14, 15, 17–25]. The majority of these patients were from Middle Eastern consanguineous families (Table 1.). Sudan has the highest percentage of consanguineous marriages in the Middle East [29]; therefore autosomal recessive forms of intellectual disabilities are expected to be prevalent compared to other modes of inheritance. The most consistent clinical presentations of patients with *TRAPPC9*-related autosomal recessive intellectual disability were cognitive impairment and delayed speech development. Some patients presented with other behavioral and metabolic abnormalities such as autism and obesity. Nonspecific dysmorphic features were reported in some but not all patients including our study. The clinical features of the two siblings in our study are aligned with those reported with TRAPPC9 mutations. More recently, missense mutations in *TRAPPC9* gene have been reported in three patients with intellectual disability and biochemical abnormalities consistent with Congenital disorder of glycosylation [17]. This underlies the clinical heterogeneity of *TRAPPC9-*related disorder.

The *TRAPPC9* gene is imprinted with predominance of the maternal allele [8]. It is highly expressed in postmitotic neurons and plays important roles in neuronal cells differentiation through regulating axon and/or dendrite outgrowth [30]. It is also involved in memory processing and regulation of food intake [8]. *Trappc9*^*−/−*^ knockout mice were obese and had reduced brain size [8]. This pleiotropy can explain the heterogeneous clinical features of patients with *TRAPPC9-*realted disorder.

## Conclusion

This is the first study to report patients with *TRAPPC9*-related disorder from Sub-Saharan Africa.

## Data Availability

The raw data generated in this study has been deposited in the European Variation Archive (EVA)[31] at EMBL-EBI under accession number PRJEB55655 (https://www.ebi.ac.uk/eva/?eva-study=PRJEB55655) and the variant has been submitted to Clinvar under accession number: VCV001683498.1.

## Abbreviations

*TRAPPC9*: Trafficking protein particle complex subunit 9
IKK: IκB kinase
NSMD: Nonsense mediated decay
SD: Standard deviation
DNA: Deoxyribonucleic acid
BWA: Burrows-Wheeler Aligner
NGS: Next generation sequencing
